# Manganese Neurotoxicity: Lessons Learned from Longitudinal Studies in Nonhuman Primates

**DOI:** 10.1289/ehp.0800035

**Published:** 2008-10-03

**Authors:** Neal C. Burton, Tomás R. Guilarte

**Affiliations:** Department of Environmental Health Sciences, Johns Hopkins Bloomberg School of Public Health, Baltimore, Maryland, USA

**Keywords:** cognitive function, dopamine, manganese, neurodegeneration, neurotoxicity, non human primates, Parkinson disease, positron emission tomography

## Abstract

**Background:**

Exposure to excess levels of the essential trace element manganese produces cognitive, psychiatric, and motor abnormalities. The understanding of Mn neurotoxicology is heavily governed by pathologic and neurochemical observations derived from rodent studies that often employ acute Mn exposures. The comparatively sparse studies incorporating *in vivo* neuroimaging in nonhuman primates provide invaluable insights on the effects of Mn on brain chemistry.

**Objectives:**

The purpose of this review is to discuss important aspects of Mn neurotoxicology and to synthesize recent findings from one of the largest cohorts of nonhuman primates used to study the neurologic effects of chronic Mn exposure.

**Discussion:**

We reviewed our recent *in vivo* and *ex vivo* studies that have significantly advanced the understanding of Mn-induced neurotoxicity. In those studies, we administered weekly doses of 3.3–5.0 (*n* = 4), 5.0–6.7 (*n* = 5), or 8.3–10.0 mg Mn/kg (*n* = 3) for 7–59 weeks to cynomolgus macaque monkeys. Animals expressed subtle deficits in cognition and motor function and decreases in the *N*-acetylaspartate-to-creatine ratio in the parietal cortex measured by magnetic resonance spectroscopy reflective of neuronal dysfunction. Impaired striatal dopamine release measured by positron emission tomography was observed in the absence of changes in markers of dopamine neuron degeneration. Neuropathology indicated decreased glutamine synthetase expression in the globus pallidus with otherwise normal markers of glutamatergic and GABAergic neurotransmission. Increased amyloid beta (A4) precursor-like protein 1 gene expression with multiple markers of neurodegeneration and glial cell activation was observed in the frontal cortex.

**Conclusions:**

These findings provide new information on mechanisms by which Mn affects behavior, neurotransmitter function, and neuropathology in nonhuman primates.

## Biological role of manganese

Manganese is an essential micronutrient that has a broad role in macromolecular metabolism. Mn plays a role in immune response, blood sugar homeostasis, adenosine triphosphate (ATP) regulation, reproduction, digestion, and bone growth (Aschner and Aschner 2005). It is a necessary component of metalloenzymes such as Mn superoxide dismutase, arginase, phosphoenol-pyruvate decarboxylase, and glutamine synthetase (GS) (Aschner and Aschner 2005). GS, an enzyme that converts glutamate into glutamine, is thought to be associated with up to 80% of brain Mn (Prohaska 1987). Mn has a heterogeneous distribution throughout the brain. In the normal human brain, Mn is most concentrated in the globus pallidus, caudate, and putamen and is less concentrated in cortical areas (Larsen et al. 1979). Intraneuronal axonal transport (Sloot and Gramsbergen 1994; Takeda et al. 1998b) and potassium-evoked ^54^Mn release in rat amygdala (Takeda et al. 1998a) demonstrate that Mn may participate in neuronal function and neuro-transmission. Mn can also enter neuronal terminals through calcium channels (Narita et al. 1990). The consumption of a Mn-deficient diet produced seizures in rats, demonstrating the importance of Mn in neuronal function (Hurley et al. 1963). Mn deficiency, although rare, can cause developmental defects including malformation of bones, altered macro-molecular metabolism, and reduced fertility (Aschner and Aschner 2005).

## Sources of human exposure

Mn is ubiquitous in the environment. It is present at 0.1% in the earth’s crust, and it is also a constituent of soil, ranging in concentrations from 40 to 900 mg/kg (Cooper 1984). It is released into the environment as a product of industrial activities, the use of the Mn-containing pesticide maneb, and through the use of methyl-cyclopentadienyl manganese tricarbonyl (MMT) as a gasoline antiknock agent (Agency for Toxic Substances and Disease Registry 2000). Mn is present in a variety of foods, with the highest concentrations found in nuts, legumes, and blueberries (Peterson and Skinner 1931). An estimated safe level of daily intake is 2–5 mg Mn (for review, see Aschner and Aschner 2005).

Increased Mn concentrations in the brain can occur from a variety of conditions, with contributions to human morbidity stemming from occupational, iatrogenic, medical, and environmental exposures. Occupational Mn exposure is well documented; for example, excessive Mn exposure occurs in occupations such as ferro alloy smelting (Bast-Pettersen et al. 2004; Kaji et al. 1993; Mergler et al. 1994), welding (Bowler et al. 2007; Josephs et al. 2005), mining (Montes et al. 2008; Rodriguez-Agudelo et al. 2006), battery assembly (Bader et al. 1999), and the manufacture of glass and ceramics (Srivastava et al. 1991).

Iatrogenic Mn exposure occurs in individuals receiving total parenteral nutrition (Alves et al. 1997) resulting in increased concentrations of Mn in the brain (Reimund et al. 2000). Further, exposure to at-risk populations with compromised or immature blood–brain barriers or underdeveloped excretory pathways, such as children, can also result in increased brain Mn levels after total parenteral nutrition (Iinuma et al. 2003). In support of this concept, Mn has been shown to accumulate in the brain of postnatal day (PN) 21 rat pups to a greater extent than PN70 young adult rats after 21 days of oral exposure to Mn (Dorman et al. 2000).

Mn is excreted primarily through the bile. Individuals with a decreased capacity for biliary Mn excretion also experience Mn neurotoxicity. Increased Mn has been measured in the blood (Spahr et al. 1996) and brains (Burkhard et al. 2003) of individuals with cirrhosis. Further, increases in brain Mn measured by magnetic resonance imaging (MRI) has been documented in patients with liver dysfunction (Fukuzawa et al. 2006; Rose et al. 1999) and patients with liver failure (Aggarwal et al. 2006; Brunberg et al. 1991; Klos et al. 2006a).

Recently, Mn exposure has also been associated with use of methcathinone (ephedrone) contaminated with the oxidant potassium permanganate added to the preparation. Ephedrone is used intravenously as a drug of abuse, and case reports suggest that the rapid onset of hypokinesia, dysarthria, dystonia, and postural dysfunction in these recreational drug users is a product of Mn neurotoxicity (de Bie et al. 2007; Holzgraefe et al. 1986; Sanotsky et al. 2007; Sikk et al. 2007).

The use of MMT as a gasoline antiknock agent has raised concerns over potential increased environmental exposures of the general population to Mn (Kaiser 2003). Elevated average indoor and outdoor air concentrations of Mn have been reported near expressways in Montreal, Quebec, Canada, which uses MMT in gasoline, compared with rural communities in which MMT use is infrequent (Bolte et al. 2004). The contribution from the use of MMT to environmental levels of Mn to humans is an important issue, as an increased environmental Mn burden as a consequence of Mn generated from MMT-containing gasoline and industrial activities may be associated with an increased prevalence of movement abnormalities and parkinsonism.

## Toxicologic role

At high levels of exposure, Mn can produce multiple symptoms of neurotoxicity. Couper (1837) first described progressive motor abnormalities in workers occupationally exposed to Mn oxide. Mn poisoning has since been associated with an extra-pyramidal syndrome including kinetic tremor, bradykinesia, rigidity, dystonia, and specific gait disturbances (Banta and Markesbery 1977; Cersosimo and Koller 2006; Huang et al. 1997). In the absence of continued exposure, motor deficits can persist for many years (Bouchard et al. 2007). In addition to these motor abnormalities, neuro psychologic symptoms including deficits in working memory, concentration, and spatial orientation also occur early in Mn neurotoxicity (Josephs et al. 2005; Klos et al. 2006b; Mergler et al. 1994). A study conducted in Bangladesh showed a significant association between exposure to high levels of Mn in well water and deficits in cognitive function assessed by verbal scores and measures of intellectual ability of children (Wasserman et al. 2006). Importantly, a diagnosis of Parkinson disease (PD) at an earlier average age was correlated with elevated ambient Mn concentrations related to industrial emissions in a Canadian study (Finkelstein and Jerrett 2007). In addition, increased air concentrations of Mn derived from mining and industrial activities in Mexico were statistically significantly associated with deficits in movement coordination and hand position changes (Rodriguez-Agudelo et al. 2006). Further, an increased prevalence of PD in a population in Brescia, Italy, was associated with increased Mn concentrations in settled dust resulting from nearby ferroalloy plants (Lucchini et al. 2007).

## Toxicokinetics

Mn is known to have 11 oxidation states (Takeda 2003), although only two oxidation states—Mn^2+^ and Mn^3+^—are common in biological systems (Archibald and Tyree 1987). Mn^2+^ is the most stable, although Mn^3+^ most efficiently oxidizes intra-cellular substrates such as dopamine (Archibald and Tyree 1987). Mn^3+^ binds to and is transported by transferrin (Aschner et al. 2007), whereas it is postulated that Mn^2+^ must use other transporter systems to enter cells (Takeda 2003). Gastrointestinal absorption and inhalation are the two main routes by which Mn is absorbed into the human body.

Approximately 1–5% of gastrointestinal Mn is absorbed (Davis et al. 1993). Sixty to 70% of inhaled Mn is expelled from the lung by mucocilliary movement and is swallowed (Mena 1974), although Mn absorption also occurs directly in the lung (Vitarella et al. 2000). Importantly, Mn can be transported directly into the brain during inhalation via the olfactory tract (Dorman et al. 2002). Mn is mainly excreted through the bile (Davis et al. 1993). Under normal conditions, Mn concentrations in the blood are between 0.8 and 2.1 μg/L (Dobson et al. 2004). After excessive Mn exposures, average blood Mn levels have been documented at 10–40 μg/L (Takser et al. 2003), but maximum reported values from one study measured blood Mn in newborns at approximately 90 μg/L and in the birthing mother approximately 150 μg/L (Takser et al. 2003). In blood, Mn is present as a free ion (Takeda 2003), and it is also bound to serum proteins and metal binding proteins such as transferrin (Aschner and Aschner 2005).

Mn enters the brain at the capillary endothelium across the choroid plexus (Rabin et al. 1993). Calcium has been shown to play an important role in the regulation of Mn transport at the blood–brain barrier (Crossgrove and Yokel 2005). Additionally, divalent metal ion transporter 1 (DMT1)-mediated transport (Au et al. 2008), the *N*-methyl-d-aspartate (NMDA) receptor channel (Itoh et al. 2008), transferrin (Aschner and Gannon 1994), diffusion, and active transport (Rabin et al. 1993) also contribute to Mn transport across the blood–brain barrier (for review, see Aschner et al. 2007). The importance of the transferrin pathway in brain uptake of Mn is underscored by observations of increased Mn levels in the brains of rats following iron deficiency, which up-regulates transferrin-mediated transport (Aschner 2000).

Once Mn has crossed the blood–brain barrier, the average half-life of Mn in the rat brain is 51–74 days (Takeda et al. 1995). In nonhuman primates, available data suggest that the rate of elimination in the brain has region specificity; however, estimates of half-life range from = 33 days (Dorman et al. 2006a) to 53 days (Newland et al. 1987) after inhalational and subcutaneous Mn exposure, respectively. The normal concentration of Mn in the brain is, on average, 0.26 μg/g wet weight (Markesbery et al. 1984), although Mn distribution throughout the brain is known to be heterogeneous. In nonhuman primates chronically exposed to Mn by intravenous injection and inhalation, Mn has been observed to accumulate throughout the brain *in vivo* using T1-weighted MRI (Dorman et al. 2006b; Guilarte et al. 2006b). The region of the brain in which Mn accumulates to the greatest extent is the globus pallidus. Using inhalation as a route of exposure, Dorman et al. (2006a) demonstrated that the globus pallidus accumulates Mn concentrations in a dose-dependent fashion and ranges from 1.6- to 6.0-fold relative to control animals after occupationally relevant exposures. An intravenous injection paradigm of Mn exposure produced an approximately 5-fold increase in pallidal Mn concentrations (Guilarte et al. 2006b).

Mn can be taken up into astrocytes and neurons. Erikson and Aschner (2006) showed that astrocytic uptake of Mn takes place at least in part via DMT1. Neuronal uptake of Mn involves transferrin (Suarez and Eriksson 1993) as well as utilization of specific transporter systems such as the dopamine transporter (DAT) (Anderson et al. 2007; Chen et al. 2006b). Intracellularly, Mn accumulates in the mitochondria (Gavin et al. 1999), and Mn accumulation in the nucleus has been demonstrated (Kalia et al. 2008). Utilization of divalent cation transporters such as the calcium uniporter (Gavin et al. 1999) and the positive valence of Mn ion may explain the localization of Mn to electron-rich mitochondria. Axonal transport of Mn occurs (Sloot and Gramsbergen 1994), which contributes to the observations of elevated concentrations of Mn efferent to iron-rich regions of the brain that have high concentrations of the transferrin receptor (Dobson et al. 2004).

Bock et al. (2008) recently compared the pharmacokinetics of Mn entry into and distribution throughout the brain in rodents and nonhuman primates as measured by T1-weighted MRI. That study demonstrated that for a given dose, Mn transport into nonhuman primate brain is greater than the absorption into the brain of rodents. Additionally, these authors demonstrated that the distribution of Mn throughout the brain differs between nonhuman primates and rodents. Bock et al. (2008) explained these differences between species as a product of Mn transport from the cerebrospinal fluid at the choroid plexus into the brain. The differences in brain anatomy between rodents and non human primates and differential positioning of brain regions such as the striatum relative to brain ventricles may drive the unique patterns of regional accumulation of Mn described in rodents and nonhuman primates. The similarities in brain structure between nonhuman primates and humans offer the opportunity to study Mn neurotoxicity in an animal model that recapitulates the pattern of human brain Mn accumulation.

## Mechanisms of Toxicity

### Mitochondrial respiration

The disruption of mitochondrial activity by Mn is well documented. Mn^2+^ uses the calcium uniporter to gain entrance into mitochondria (Gavin et al. 1999). In the mitochondria, the bulk of Mn is bound to the inner mitochondrial membrane or matrix proteins (Gavin et al. 1999). This allows Mn to directly interact with proteins involved in oxidative phosphorylation. Mn has been shown *in vitro* to interfere with ATP synthesis (Gunter et al. 2006), particularly by inhibiting the function of the F_1_ATPase (Gavin et al. 1992) and at higher concentrations by inhibiting complex I (Chen et al. 2001). Importantly, the speciation of Mn is a critical determinant of its interactions. Trivalent Mn inhibits complex I more potently than does divalent Mn (Chen et al. 2001), and Mn^3+^ more efficiently oxidizes intra cellular substrates such as dopamine (Archibald and Tyree 1987). However, divalent Mn is the predominant species found in tissue (Gunter et al. 2006). Mn has been associated with suppression of ATP-dependent calcium waves in astrocytes, suggesting that Mn promotes the potentially disruptive mitochondrial sequestration of calcium (Tjalkens et al. 2006). Nuclear magnetic resonance spectroscopy has been used in culture to show that Mn decreases ATP/adenosine diphosphate ratios and impairs glucose metabolism and metabolic activity in the globus pallidus (Zwingmann et al. 2007).

### Oxidative stress

The generation of reactive oxygen species is intimately linked to mitochondrial activities, and there is much evidence to support the role of reactive oxygen species in Mn neurotoxicity. Rats treated with Mn for 7 days by oral gavage showed increased striatal concentrations of ascorbic acid and gluta thione (GSH), antioxidants that when increased signal the presence of an elevated burden from reactive oxygen species (Desole et al. 1994). A later study conducted in rats exposed to Mn by inhalation confirmed the presence of markers of oxidative stress, with decreased GSH and increased metallothionine in the Mn-exposed animals (Dobson et al. 2003). Nonhuman primate studies further substantiated the association between Mn exposure and increases in metallothionine and decreases in GSH (Erikson et al. 2007). Addition of Mn to cultured astrocytes increases 2′,7′-dichlorofluoroscein fluorescence indicative of increased reactive oxygen species, and coincubation of *N*-acetylcysteine blocks Mn toxicity (Chen and Liao 2002).

Not only has Mn exposure been associated with markers of oxidative stress, but activation of signaling pathways involved in the response to oxidative stress has been documented as well. In PC12 cells, Mn induced the expression of nuclear factor kappa B (NFκB) (Ramesh et al. 2002) and AP-1 (Wise et al. 2004), further providing support for an oxidative basis in Mn neurotoxicity. Pretreatment with either vitamin E or a NFκB inhibitor has been shown to protect against Mn toxicity in mesencephalic cells (Prabhakaran et al. 2008). The intracellular consequences of Mn-induced increases in oxidative stress are becoming clearer as we learn more about the mechanisms of Mn neurotoxicity. Recent studies have shown that in dopaminergic cells, Mn can promote apoptosis by a caspase 3–dependent activation of protein kinase C delta (Latchoumycandane et al. 2005). Mn neurotoxicity may involve oxidative stress and apoptosis, interconnected pathways that have been linked to the pathophysiology of neurodegenerative disease (Tansey et al. 2007). Microarray data from the frontal cortex of Mn-exposed nonhuman primates shows that the expression of genes involved in apoptosis and inflammation is increased relative to control animals (Guilarte et al. 2008a).

### Dopaminergic synapses

Much research has endeavored to describe the mechanisms by which Mn produces adverse motor function outcomes reminiscent of idiopathic PD. Because Mn-exposed individuals often exhibit motor symptoms such as kinetic tremor and dystonia (Cersosimo and Koller 2006) not typically associated with PD and because Mn-exposed individuals generally do not respond to levodopa therapy (Lu et al. 1994), the gold standard for PD treatment, it is believed that Mn neurotoxicity involves pathologic mechanisms distinct from those of PD. Available human autopsy data suggest that the nigrostriatal system is not degenerated after Mn exposure (Perl and Olanow 2007). Mn may cause dysfunction of the dopamine neuronal terminal that may set the stage for susceptibility to a later insult. However, the evidence for Mn-induced dopaminergic neuronal dysfunction is incomplete. It is known that *in vitro* Mn can promote autooxidation of dopamine, which leads to the creation of reactive dopa mine quinones (Miller et al. 1990; Shen and Dryhurst 1998). However, early rodent data offer conflicting evidence on the influence of Mn exposure on catecholamine concentrations (Bonilla and Prasad 1984; Chandra and Shukla 1981; Eriksson et al. 1987a; Gianutsos and Murray 1982; Komura and Sakamoto 1994). In nonhuman primates, high cumulative exposures to Mn > 300 mg/kg show reduced striatal concentrations of dopamine (Bird et al. 1984; Eriksson et al. 1987b; Neff et al. 1969); however, studies using lower doses have demonstrated no effect of Mn on dopamine levels (Olanow et al. 1996; Struve et al. 2007). Additional evidence from nonhuman primate data suggests a Mn-induced post synaptic decrease of D2-like dopamine receptor levels (Eriksson et al. 1992). However, the limited number of studies that have examined the effect of Mn in the context of chronic exposure leave the relationship between Mn, the dopaminergic system, and the development of motor dysfunction an open question.

### Glutamatergic synapses

There is a persistent notion in the literature that a component of Mn neurotoxicity may be modified by dys regulation of excitatory glutamatergic neurotransmission. Increased glutamate levels in the brain have been documented in rodents exposed to Mn (Gwiazda et al. 2002; Lipe et al. 1999; Reaney et al. 2006). Zwingmann et al. (2007) showed that Mn increases glutamate in some areas such as the frontal cortex but decreases it in the globus pallidus, but additional research suggests that glutamate levels are not affected by Mn exposure in rodents (Bonilla et al. 1994). These inconsistencies are most likely a consequence of varied Mn doses, routes of administration, dosing intervals and durations, species-based differences in pharmacokinetics, and differences in the brain regions analyzed. *In vitro* studies have implicated Mn in the reduction of glutamate transporter expression (Erikson and Aschner 2002; Mutkus et al. 2005) and astrocytic glutamate uptake (Hazell and Norenberg 1997). These alterations could negatively impact synaptic glutamate metabolism and distribution. Additional limited data are available on the effect of chronic Mn exposure in nonhuman primates and its effects on the glutamatergic systems. However, available evidence suggests that nonhuman primates exposed to Mn via inhalation for 13 weeks did not have altered glutamate levels in the globus pallidus, caudate, and putamen (Struve et al. 2007) but did express increased mRNA expression and decreased protein levels of GLAST and GLT, the two main astrocytic glutamate transporters, and GS, the enzyme that converts glutamate into glutamine, in multiple brain regions (Erikson et al. 2007).

### GABAergic synapses

Evidence for the involvement of GABAergic systems in Mn neurotoxicity stems from early observations that Mn exposure in rodents increases striatal GABA (γ-aminobutyric acid) concentrations (Bonilla 1978). Later studies showed that chronic Mn administration to rodents produced an increase in striatal GABA concentrations (Gianutsos and Murray 1982) and abolished age-dependent declines in glutamic acid decarboxylase (Lai et al. 1981), the enzyme that converts glutamate to GABA. Additional rodent studies further supported an association between Mn exposure and increased brain GABA concentrations (Gwiazda et al. 2002; Lipe et al. 1999; Reaney et al. 2006). However, other rodent studies complicated this picture by showing that Mn decreases striatal and frontal cortex GABA levels (Brouillet et al. 1993; Seth et al. 1981). Moreover, other rodent studies show no association between GABA levels and Mn exposure (Bonilla et al. 1994). Little information is available regarding the GABAergic system in the context of Mn exposure in nonhuman primates. One study available in rhesus monkeys chronically exposed to Mn by inhalation indicates that in multiple brain regions Mn does not alter GABA levels (Struve et al. 2007). Another study that used monthly injections of Mn oxide in nonhuman primates for 2 years showed no effect of Mn on GABAa receptor levels (Eriksson et al. 1992).

### Glial cells

Accumulating evidence indicates that Mn may indirectly affect neuronal function by damaging glial cells. Autopsy data from humans exposed to Mn indicate the presence of astrocyte activation in the brain (for review, see Perl and Olanow 2007). These associations have been recapitulated in animal models. Astrogliosis and Alzheimer type II astrocytes have been demonstrated in nonhuman primates treated acutely (Pentschew et al. 1963) and chronically (Guilarte et al. 2008a; Olanow et al. 1996) with Mn. In cultured astrocytes treated with Mn, cellular swelling was observed within 24 hr of Mn exposure (Rama Rao et al. 2007). Further, Mn disrupts intracellular calcium homeostasis and intercellular calcium waves in primary astrocytes (Tjalkens et al. 2006). Increased inducible nitric oxide synthase (iNOS) mRNA and protein and increased release of nitric oxide have also been observed after Mn exposure in primary astrocytes (Spranger et al. 1998), microglial cells (Bae et al. 2006), mesencephalic cells (Prabhakaran et al. 2008), and Mn-exposed mice (Liu et al. 2006). The nitric oxide pathway is intimately linked with NMDA receptor-mediated excitotoxic processes and microglial activation (Dawson et al. 1991; Hewett et al. 1994). One group has demonstrated in cultured astrocytes (Erikson and Aschner 2002), in rodents (Erikson et al. 2004), and in nonhuman primates (Erikson et al. 2007) that Mn exposure causes a decrease in astrocytic glutamate transporters and the astrocytic enzyme GS.

Microglia are also affected by Mn exposure. Microglial cells treated with Mn readily release hydrogen peroxide (Zhang et al. 2007). In rats treated acutely with Mn, Hazell et al. (2003) observed an increase in the trans locator protein (18 kilodaltons) (TSPO-18kDa) (previously known as the peripheral benzodiazepine receptor). In the brain, TSPO-18kDa is a microglial and astrocytic protein that has been used as a marker of gliosis and is useful in identifying areas of brain injury and inflammation (Chen and Guilarte 2008). An increase in proinflammatory genes such as tumor necrosis factor-α, iNOS, and activated inflammatory proteins such as P-p38, P-ERK, and P-JNK have been measured in primary rat glial cells after Mn exposure (Chen et al. 2006a). Mn also potentiates lipopolysaccharide-induced increases in proinflammatory cytokines in microglial cultures (Filipov et al. 2005) and increases in nitric oxide production (Chang and Liu 1999). This association between Mn exposure and inflammatory gene expression is supported by microarray data from the frontal cortex of Mn-exposed nonhuman primates that show that chronic Mn exposure increases of the expression of inflammatory genes such as osteopontin and the interferon-γ receptor (Guilarte et al. 2008a).

## Chronic Mn Neurotoxicity in Nonhuman Primates

Much of the available data that inform our understanding of the mechanisms of Mn neurotoxicity is derived from rodent studies. Little work has been comprehensively conducted analyzing the effects of chronic Mn exposure in nonhuman primates. We have analyzed one of the largest cohorts of nonhuman primates ever used to study chronic Mn neuro toxicity. Experiments are still under way; however, the available data thus far support the involvement of dopaminergic nigrostriatal dysfunction, selective astrocytic modulation of glutamate processing in the globus pallidus, and multiple indices of neurotoxicity in brain regions not generally associated with Mn exposure. Here, we summarize the findings from behavioral, neuroimaging, and neuro pathologic studies that provide important insights on mechanisms suggested by previous studies and more pathways not previously thought to be operational in Mn neuro toxicity. The average blood Mn concentrations of the animals we used were in the 65–85 μg/L range (Guilarte et al. 2006a, 2008b). This concentration is within the upper range of blood Mn concentrations documented in human populations such as those living near Mn mining areas (Santos-Burgoa et al. 2001), in mothers and newborns (Takser et al. 2003) and in children in communities in which MMT is used in gasoline (Gulson et al. 2006), and as a result of parenteral nutrition (Iinuma et al. 2003).

### Cognition and behavior

Because humans exposed to high levels of Mn experience behavioral abnormalities (Mergler and Baldwin 1997) and cognitive deficits (Lucchini et al. 1995), we sought to characterize the involvement of behavioral and cognitive disturbances in nonhuman primates exposed to 3.3–5.0 mg Mn/kg/week for approximately 10 months (Schneider et al. 2006). Animals (cynomolgus macaques) were trained on a series of tasks that measure behavior and cognitive performance. After approximately 20 weeks of exposure and continuing throughout the duration of the study, the Mn-exposed animals developed a small but statistically significant increase in the rate of PD-like behaviors on a scale developed to assess parkinsonian behaviors in non human primates (Schneider and Kovelowski 1990). Fine motor skills were assessed by an easy task and by a difficult task. Although Mn exposure had no effect on the easy task, by the end of the observation period the animals performed poorly on the difficult task. These observations are consistent with documentation of humans exposed to Mn occupationally or as a consequence of treatment with total parenteral nutrition who suffer cognitive deficits (Josephs et al. 2005; Klos et al. 2006b; Mergler et al. 1994) and also perform poorly on tests of fine motor ability (Shin et al. 2007). The Mn-exposed animals also expressed stereotypic and compulsive-like behaviors that may be explained by involvement of dysfunctional frontostriatal circuitry.

### Magnetic resonance spectroscopy

Few data are available that comprehensively characterize Mn neurotoxicity throughout the brain, especially in the context of chronic exposure in nonhuman primates. Therefore, we used ^1^H-magnetic resonance spectroscopy (^1^H-MRS) to longitudinally assess the potential toxic effects of chronic Mn exposure on levels of brain metabolites (Guilarte et al. 2006b) that are thought to reflect various aspects of neuronal and glial functioning in the brain (Jenkins and Kraft 1999). The concentrations of the brain metabolites creatine, *N*-acetylaspartate (NAA), choline, and myoinositol were characterized by ^1^H-MRS in the parietal cortex, striatum, thalamus, and frontal white matter. The globus pallidus could not be assessed because the iron content interferes with acquisition of the ^1^H-MRS signal. Data were normalized to creatine content in a particular brain region. NAA is a neuron-specific metabolite, myoinositol is used as a glial cell marker, and choline is a substituent of cell membranes (De Stefano et al. 2007). Reductions in NAA/creatine are reflective of neuronal loss and/or dysfunction, alterations in myoinositol/crea tine are suggestive of glial activation or damage, and modulation of choline/creatine can provide important information on membrane phospholipids in the context of myelin breakdown. Whereas the ratio of choline/crea tine and myoinositol/creatine were unchanged by chronic Mn exposure, the ratio of NAA/creatine was significantly reduced in the parietal cortex of Mn-exposed animals relative to baseline. A near-significant (*p* = 0.055) decrease was observed in frontal white matter. Reduced NAA levels also occur in pathologic conditions such as stroke and Alzheimer’s disease (Kantarci 2007), and the reduction of NAA is reflective of neuronal dysfunction or degeneration. The *in vivo* analy sis of Mn-exposed animals by MRS further underscores observations that although Mn accumulates to the greatest extent in the globus pallidus, other regions of the brain that may accumulate Mn to a lesser extent can still be vulnerable to Mn neurotoxicity.

### Dopaminergic synapses

Given the similarities in some of the symptoms of Mn intoxication and the clinical symptoms of PD, as well as available supporting animal research implicating the dopaminergic system in Mn neurotoxicity, we have characterized multiple aspects of the dopaminergic synapse in nonhuman primates exposed to Mn both acutely and chronically using positron emission tomography (PET) and *ex vivo* methods.

In an early study (Chen et al. 2006b), we examined the effect of acute Mn exposure on DAT levels in baboons as assessed by PET and post mortem analysis. Acute Mn exposure caused a transient increase in DAT levels within 1 week of exposure that normalized after approximately 1 month. Further, it was shown that Mn inhibits [^3^H]-dopamine uptake in rat striatal synaptosomes. Although Mn has no effect on the affinity (*K*_d_) of the cocaine analog [^3^H]-WIN 35,428 binding to DAT, we observed a statistically significant 30% reduction of the *B*_max_, indicating that although Mn has no effect of the affinity of the DAT ligand, it reduces the available number of binding sites. This suggested that Mn may affect the presynaptic dopaminergic neuronal terminal through modulation of DAT function and levels.

We have also used neuroimaging and neuropathologic techniques to comprehensively probe the effect of chronic Mn exposure in cynomolgus macaques on multiple parameters of the dopaminergic synapse (Guilarte et al. 2006a, 2008b). The most dramatic change observed with chronic Mn exposure was a reduction in the capacity of striatal dopamine release, measured by PET with amphetamine (AMPH) challenge. Importantly, an animal receiving the highest dose of Mn (8.3–10.0 mg/kg/week) that expressed dystonia and dyskinesias also exhibited the largest decline in AMPH-induced dopa mine release from a baseline of 44% to 8.0% release after only 7 weeks of Mn exposure (Guilarte et al. 2008b). These findings indicate that Mn-induced motor function deficits are caused by mechanisms that inhibit dopamine release in the presence of normal levels of dopa mine and an intact nigrostriatal dopa mine system. This view is in contrast to theories suggesting that Mn neurotoxicity is mediated by an effect on post-synaptic dopamine receptors.

Importantly, the use of control animals that received no Mn but did receive repeated AMPH injections as part of the PET imaging protocol provided insight on the activity of Mn at the dopaminergic synapse. Although AMPH administration was necessary to characterize the *in vivo* capacity for dopamine release by PET, AMPH itself causes alterations in the integrity of dopaminergic neuronal terminals. However, this image-control group showed greater deficits in DAT, vesicular monoamine transporter type 2, and dopamine and its metabolites relative to Mn-exposed animals that received equivalent amounts of AMPH normalized to body weight. This suggests that Mn may directly interact with DAT and interfere with AMPH uptake into dopaminergic neurons. This is consistent with previously published reports that Mn is a substrate for DAT (Anderson et al. 2007; Ingersoll et al. 1999) and that Mn inhibits binding of a cocaine analog to DAT and inhibits dopamine uptake in striatal synaptosomes (Chen et al. 2006b). These findings strongly suggest that the documented motor function abnormalities in Mn-exposed nonhuman primates at Mn doses given in this study are mediated by inhibition of dopa mine release in the striatum and shed new light on the molecular basis of Mn-induced parkinsonism.

### Glutamatergic/GABAergic synapses

Evidence of the involvement of the glutamate and GABA neurotransmitter systems can be traced back three decades and involves studies conducted in primary cell culture models, rodents, and nonhuman primates. However, the culture and rodent data has been contradictory and inconclusive (see “Glutamatergic synapses” and “GABAergic synapses” above). Until recently, little information has been available in nonhuman primates on glutamate and GABA neuro trans mission throughout the brain. Therefore, we also recently studied the effect of Mn on glutamatergic and GABAergic systems throughout the nonhuman primate brain. Chronic Mn exposure did not alter glutamate, GABA, or the glycine (a coagonist for NMDA glutamate receptors) levels in frontal white matter, globus pallidus, caudate, putamen, hippocampus, thalamus, cortex (frontal, motor, somatosensory, parietal, visual), cerebellar lobules, and deep cerebellar nucleus (Burton NC, unpublished data). These findings are consistent with a recent report that suggested that chronic Mn exposure in nonhuman primates does not alter the concentration of glutamate or GABA in the globus pallidus, caudate, or putamen (Struve et al. 2007). In our recent study (Burton NC, unpublished data), Mn did not consistently promote a change in levels of NMDA receptors, GABAa receptors, or glutamate transporters. However, Mn did cause a reduction in GS in the globus pallidus, a glial-specific enzyme that converts glutamate into glutamine as part of the glutamate recycling process. The decrease in GS levels in Mn-exposed nonhuman primates is consistent with observations made in another cohort of nonhuman primates exposed to Mn via inhalation (Erikson et al. 2007). Reductions in GS levels can cause dysregulation of the glutamate recycling pathway, and by mass action, a reduction in the conversion of glutamate into glutamine could lead to an increase in intracellular glutamate concentrations in astrocytes and a potential spillover into the synapse with the potential to cause excitotoxicity in the globus pallidus.

### Frontal cortex

The neurologic sequelae of exposure to high levels of Mn occur in multiple stages. The early phase of Mn intoxication involves a psychiatric component, characterized in part by irritability, apathy, and psychosis, and is sometimes called Mn mania (Mergler and Baldwin 1997). Deficits in measures of executive function, such as short-term memory and computational ability, are also early manifestations of Mn neurotoxicity (Lucchini et al. 1995). Studies show that Mn accumulates in the frontal cortex (Bock et al. 2008; Dorman et al. 2006a; Guilarte et al. 2008a), and it has been suggested that Mn may alter cognitive domains mediated by the frontal cortex and subcortical structures (Josephs et al. 2005). Toward this end, we performed microarray analysis of frontal cortex tissue from naïve control animals and animals exposed to 3.3–5.0 mg Mn/week (Guilarte et al. 2008a). The results indicated that Mn promoted modulation of genes related to inflammation, apoptosis, cell cycle, protein turnover and folding, synaptic function, and cholesterol homeostasis. These observations suggested that Mn may promote inflammation and cell death in the frontal cortex. Further, the activation of cell cycle machinery in post-mitotic cells such as neurons disrupts normal cellular processes and has been implicated in dysfunction and cell death in Alzheimer’s disease (Raina et al. 2000; Yang et al. 2003). An imbalance in cholesterol homeostasis is associated with the development of pathologic processes present in Alzheimer’s disease (Puglielli et al. 2003). In addition, the most highly up-regulated gene was amyloid beta (A4) precursor-like protein 1 (*APLP1*), which is a member of the amyloid precursor protein involved in copper homeostasis and the regulation of the toxic peptide beta amyloid (Aβ)_1–42_ (Neumann et al. 2006). The increase in *APLP1* gene expression was confirmed by immunohistochemistry and led to the further analysis of A β levels in the frontal cortex. Unexpectedly, in the juvenile animals used in this study, extracellular diffuse aggregation of A β was observed in the frontal cortex of Mn-exposed animals, a condition normally observed in the aged brain. This suggested that Mn accelerates the aging process and promotes Alzheimer’s-like neuropathology in the frontal cortex. The presence of neurodegenerative changes in the frontal cortex of Mn-exposed animals was confirmed by the presence of neuronal agyrophilic inclusions using silver staining and by morphologic changes consistent with apoptotic stigmata (Guilarte et al. 2008a). Neurons of the frontal cortex of Mn-exposed animals expressed hypertrophic nuclei and vacuolar cell bodies suggestive of ongoing pathology. Hydroxynonenol staining, a marker of lipid peroxidation, was associated with apoptotic cells. Astrogliosis was also observed in the Mn-exposed animals. *APLP1* is regulated by the tumor suppressor p53 (Tang et al. 2007), as are many of the genes identified in the microarray analysis. Increased p53 and APLP1 staining colocalized with cells showing morphologically apoptotic features, suggesting the possibility that Mn may function through p53 to affect changes in APLP1 that promote extracellular A β accumulation. In addition, Mn exposure also caused an increase of approximately 30% in copper concentrations in the frontal cortex (Guilarte et al. 2008a). Accumulation of brain copper has been associated with A β plaques in the AD brain (Bush 2003). Dysregulation of copper homeostasis may be one additional mechanism by which Mn promotes neurodegenerative processes. Further studies are warranted in an appropriate cell culture system to determine the mechanistic relationship among Mn exposure, p53 levels and activity, APLP1, A β, and copper homeostasis. The gene expression changes and markers of neurodegeneration in the frontal cortex may help to explain not only the subtle cognitive deficits previously characterized in these Mn-exposed animals, but also some of the early manifestations of Mn neurotoxicity in humans related to working memory and neuropsychiatric behaviors.

## Conclusion

Much of the research that has informed the understanding of the mechanisms of Mn neurotoxicity is based on data generated from cell culture and research conducted in rodents. Possible mechanisms suggested by rodent data must be confirmed in higher species, especially in light of recent pharmacokinetic studies suggesting that brain Mn uptake and distribution may be dramatically different between primates and rodents (Bock et al. 2008). Unfortunately, studies conducted in nonhuman primates are few. The multidisciplinary study described within is the largest cohort of nonhuman primates that has been used to characterize the effects of chronic Mn exposure throughout the brain on neuroimaging, pathology, multiple neurotransmitter systems, behavior, and cognition. These data provide corroborative support that chronic Mn exposure does not alter total levels of dopamine, glutamate, or GABA. However, Mn may reduce GS levels and produce a glial-specific effect on the glutamatergic system in the globus pallidus by interfering with the recycling of glutamate, which may ultimately promote a redistribution of glutamate at the synapse. Additionally, PET studies demonstrated that Mn produces its most substantial effect on the nigrostriatal dopamine system not by producing its degeneration, but by disrupting the capacity to release dopamine. Last, MRS documented neuronal loss and/or dysfunction in the parietal cortex, and postmortem analysis showed neurodegeneration in the frontal cortex. These cortical regions were previously unsuspected to be involved in Mn neurotoxicity because they accumulate relatively low levels of Mn compared with the globus pallidus. These findings indicate that Mn neurotoxicity is not simply defined by the amount of Mn that a brain region accumulates but by the intrinsic vulnerability to injury by Mn.

Therefore, the continuum of dysfunction caused by Mn from psychiatric to cognitive to motor abnormalities cannot be explained by dysregulation of basal ganglia structures alone. These findings suggest that historical views of Mn neurotoxicity should be revisited to account for the spectrum of Mn-induced neurologic dysfunction.
